# Lipid Antioxidant and Galactolipid Remodeling under Temperature Stress in Tomato Plants

**DOI:** 10.3389/fpls.2016.00167

**Published:** 2016-02-17

**Authors:** Livia Spicher, Gaetan Glauser, Felix Kessler

**Affiliations:** ^1^Laboratory of Plant Physiology, Institute of Biology, University of NeuchâtelNeuchâtel, Switzerland; ^2^Neuchâtel Platform of Analytical Chemistry, University of NeuchâtelNeuchâtel, Switzerland

**Keywords:** lipidomics, prenylquinones, photosynthesis, temperature stress

## Abstract

Increased temperatures are a major scenario in climate change and present a threat to plant growth and agriculture. Plant growth depends on photosynthesis. To function optimally, the photosynthetic machinery at the thylakoid membrane in chloroplasts continuously adapts to changing conditions. Here, we set out to discover the most important changes arising at the lipid level under high temperature (38°C) in comparison to mild (20°C) and moderately cold temperature (10°C) using a non-targeted lipidomics approach. To our knowledge, no comparable experiment at the level of the whole membrane system has been documented. Here, 791 molecular species were detected by mass spectrometry and ranged from membrane lipids, prenylquinones (tocopherols, phylloquinone, plastoquinone, plastochromanol), carotenoids (β-carotene, xanthophylls) to numerous unidentified compounds. At high temperatures, the most striking changes were observed for the prenylquinones (α-tocopherol and plastoquinone/-ol) and the degree of saturation of fatty acids in galactolipids and phosphatidyl ethanolamine. Photosynthetic efficiency at high temperature was not affected but at moderately cold temperature mild photoinhibition occurred. The results indicate, that the thylakoid membrane is remodeled with regard to fatty acid saturation in galactolipids and lipid antioxidant concentrations under high temperature stress. The data strongly suggest, that massively increased concentrations of α-tocopherol and plastoquinone are important for protection against high temperature stress and proper function of the photosynthetic apparatus.

## Introduction

Plants have the ability to acclimate to changing environmental conditions. However, long-term climate change driven by rising temperatures may have a deleterious impact on plant physiology and in turn negative effects on crop yields. Plant growth is directly dependent on photosynthesis, that takes place in the chloroplast. The chloroplast contains an extensive membrane system, the thylakoids that harbor the photosynthetic machinery responsible for the light reactions.

The thylakoid membrane consists mostly of galactolipids, composed of 50% of monogalactosyldiacylglycerol (MGDG) and 26% digalactosyldiacylglycerol (DGDG), phosphatidylglycerol, and sulfoquinovosyldiacylglycerol making up most of the remainder. The abundance of galactolipids in the photosynthetic membranes suggests they have not only typical bilayer functions but also specific roles, such as the stabilization of photosynthetic complexes, membrane architecture (curvature), thylakoid stack (grana) formation (Dörmann, 2013).

In addition to membrane lipids, the thylakoids contain embedded lipid antioxidants such as tocopherols, plastochromanol as well as plastoquinone (that is better known as an electron transporting redox molecule; Munné-Bosch and Alegre, 2002; Gruszka et al., 2008; Mène-Saffrané and DellaPenna, 2010; Nowicka and Kruk, 2012; Rastogi et al., 2014; Ksas et al., 2015).

Chloroplasts and photosynthetic membranes respond to environmental changes. They acclimate to intensity and quality of light, with changes in the structure of thylakoid membranes, the size of grana stacks, chlorophyll content, amount, and localization of light harvesting complexes (LHCII), to mention some (Kanervo et al., 1997; Rochaix, 2007; Lichtenthaler, 2010; Rochaix et al., 2012). The modification of the saturation level of membrane lipids is an important acclimation strategy of plants in response to temperature changes, allowing to maintain parameters such as membrane fluidity and permeability in response to varying temperature (Zheng et al., 2011). Recent findings also reported that reversible increase in the size of chloroplasts and number of plastoglobules occurs under moderate temperature stress (Zhang et al., 2010).

Upon abiotic stress, such as high or low temperature and high-light, the homeostasis of reactive oxygen species (ROS) metabolism in the plant cell is challenged (Lichtenthaler and Burkart, 1999; Apel and Hirt, 2004; Zhang et al., 2010). A protective system in part based on lipid soluble molecules is in place to protect plant cells and the photosynthetic membranes against the action of ROS. Carotenoids— such as β-carotene, lutein, neoxanthin, and three xanthophyll cycle carotenoids, zeaxanthin, violaxanthin, and antheraxanthin,—besides structural stabilization and light harvesting roles, play important photoprotective roles in scavenging of singlet oxygen species and quenching of chlorophyll triplet states, and in excess energy dissipation by non-photochemical quenching (NPQ) that implicates xanthophylls and lutein (Mimuro and Katoh, 1991; Frank and Cogdell, 1996; Choudhury and Behera, 2001; Gruszecki and Strzalka, 2005; Shumskaya and Wurtzel, 2013). Another category of molecules, the prenylquinones (including tocopherols, plastoquinone, plastochromanol) are lipid soluble compounds, that function as membrane-protective antioxidant molecules. Tocopherols have widely been described as antioxidants against high-light triggered oxidative stress (Havaux et al., 2005; Krieger-Liszkay and Trebst, 2006; Kobayashi and DellaPenna, 2008). Plastoglobule-localized enzymes are actively implicated in the lipid metabolic pathways during high-light stress: NAD(P)H-dependent quinone oxidoreductase (NDC1), and tocopherol cyclase (VTE1) participate in the synthesis and the recycling of tocopherols and plastochromanol (PC-8). In addition, NDC1 is essential for phylloquinone production (Eugeni Piller et al., 2011; Besagni and Kessler, 2013; Fatihi et al., 2015).

Therefore, the content of carotenoids and prenylquinones changes in response to stress as well as throughout the development stages of the plant. These changes can be seen as an integral part of the lipid remodeling taking place at the thylakoid membrane system. This paper analyses changes in lipid composition during temperature stress using liquid chromatography-mass spectrometry based lipidomics methods. Lipidomics are a powerful tool set enabling identification and fingerprinting of different classes of lipids, ranging from galactolipids and their degree of fatty acid saturation, to antioxidant prenylquinones and carotenoids.

The study was carried out in tomato (*Solanum lycopersicum* L.). Even though it is a species of tropical origin, it is cultivated worldwide and has become an important agricultural crop. Therefore, tomato is a relevant model system to study how variation in temperature affects the lipid composition in leaves.

## Materials and methods

### Plant material and stress treatments

Tomato plants (*S. lycopersicum* L.) variety M82, grown on soil under conditions referred to as control (250 μmol.m^−2^.s^−1^ of light, 16-h light/8-h dark, at 20/18°C, with 55% relative air humidity). 5 to 6-week old plants were transferred to either 10/8°C (day/night), 38/30°C, or remained in 20/18°C, as control conditions. After 6 days, plants that were all returned to control conditions for a recovery period of 5 days.

### Determination of photosynthetic parameters

Maximum photochemical efficiency or optimum quantum yield of photosystem II (*F*v/*F*m), was fluorometrically determined using a MINI-PAM Photosynthesis Yield Analyzer (Walz, http://www.walz.com). Plants were dark-adapted for 15 min before *F*v/*F*m measurements with illumination by application of a saturation flash. Five replicates for each treatment were done, at day 0 and on days 2, 4, 6, and 11 days of temperature treatments. The data obtained from *F*v/*F*m measurements in leaves were subjected to One-Way analysis of variance (One-Way ANOVA), followed by Bonferroni–Holm test to determine any significant differences in photosynthetic efficiency (Aickin and Gensler, 1996).

### Chlorophyll quantification

Chlorophyll was extracted from 100 mg of tissue in 1 ml of 80% acetone. Total chlorophyll levels were then spectrophotometrically determined (Inskeep and Bloom, 1985). Five replicates for each treatment were done, at days 0, 6, and 11 of the temperature treatments. The data obtained from the measurements were subjected to One-Way ANOVA, followed by Bonferroni–Holm test to determine any significant differences between the various temperatures over the time course.

### Lipid profiling

Samples were prepared and analyzed from the five replicates for each treatment, at days 0, 6, and 11 of the temperature treatments, according to Martinis et al. (2013) with small modifications. In brief, lipids were extracted from 100 mg of fresh tissue and suspended in 1 ml of tetrahydrofuran:methanol 50:50 (v/v). 5–10 glass beads (1 mm in diameter) were added to the mixture and homogenized for 3 min at 30 Hz in a tissue lyser. After centrifugation (3 min, 14,000 g, and 4°C), the supernatant was transferred to an HPLC vial. Lipid profiles were obtained by ultra-high pressure liquid chromatography coupled with atmospheric pressure chemical ionization-quadrupole time-of-flight mass spectrometry (UHPLC-APCI-QTOF-MS) as described in Martinis et al. (2011, 2013). Separation was performed on a reverse-phase Acquity BEH C18 column (50 × 2.1 mm, 1.7 μm) under the following conditions: solvent A = water; solvent B = methanol; 80–100% B in 3 min, 100% B for 2 min, re-equilibration at 80% B for 0.5 min. The flow rate was 0.8 ml min^−1^ and the injection volume was 2.5 μl. Data were acquired using MassLynx version 4.1 (Waters), and further processed with MarkerLynx XS (Waters) to generate peak lists consisting of variables described by mass-to-charge ratio and retention time. Multivariate analysis was carried out using the statistics softwares EZinfo and Simca v.13.0.3 (Umetrics). Variables were Pareto-scaled before applying principal component analysis (PCA) and supervised partial least square discriminant analysis (PLS-DA; Eugeni Piller et al., 2011; Martinis et al., 2011). In Pareto scaling, variables are divided by the square-root of their standard deviation as an intermediate between no scaling and dividing variables by their standard deviation alone. PLS-DA is a supervised multivariate method which takes advantage of class information. For PLS-DA models, the predictive ability and the degree of overfitting were evaluated using a leave-one-subject-out cross-validation and permutation tests with 200 random permutations. R2 and Q2 coefficient values were calculated for the original and permuted models. Identification of the variables of interest was achieved through comparison with pure standards whenever available. When standards were not available, tentative identification was performed by combining determination of elemental compositions (with accurate mass and isotopic ratios provided by QTOF-MS), fragmentation by collision induced dissociation to obtain characteristic fragments, and search in online databases such as LIPID MAPS (http://www.lipidmaps.org/data/structure/LMSDSearch.php?Mode=SetupTextOntologySearch) and PUBCHEM (https://pubchem.ncbi.nlm.nih.gov/search/search.cgi#). For complete information regarding the identification procedure refer to Supplemental Table 1. For an example of the identification procedure refer to Supplemental Figure 1. In addition to untargeted analysis, the UHPLC-APCI-QTOFMS method enabled the absolute quantification of several lipids for which reference standards were available. Absolute concentrations of δ-T, γ-T, α-T, α-TQ, PQ-9, PC-8, and phylloquinone (Vit K) were measured based on calibration curves obtained from pure standards. Moreover, PC-OH and PQ-OH were quantified as PC-8 and PQ-9 equivalents, respectively. Tocopherol and phylloquinone standards were obtained from Sigma-Aldrich, and PC-8 and PQ-9 standards were kindly provided by Kruk (Kruk, 1988; Gruszka and Kruk, 2007). The other molecules identified for which pure standards were unavailable were quantified relatively based on peak intensity measurements in the chromatograms. The two carotenoids violaxanthin and neoxanthin were measured as a sum since they could not be resolved either in the chromatographic or the mass dimensions under the conditions employed. The data obtained from the measurements were subjected to One-Way ANOVA, followed by Bonferroni–Holm test to determine any significant differences between different temperatures over the time course.

## Results

### Photosynthetic efficiency is reduced after cold temperature treatment

**Figure 1:**
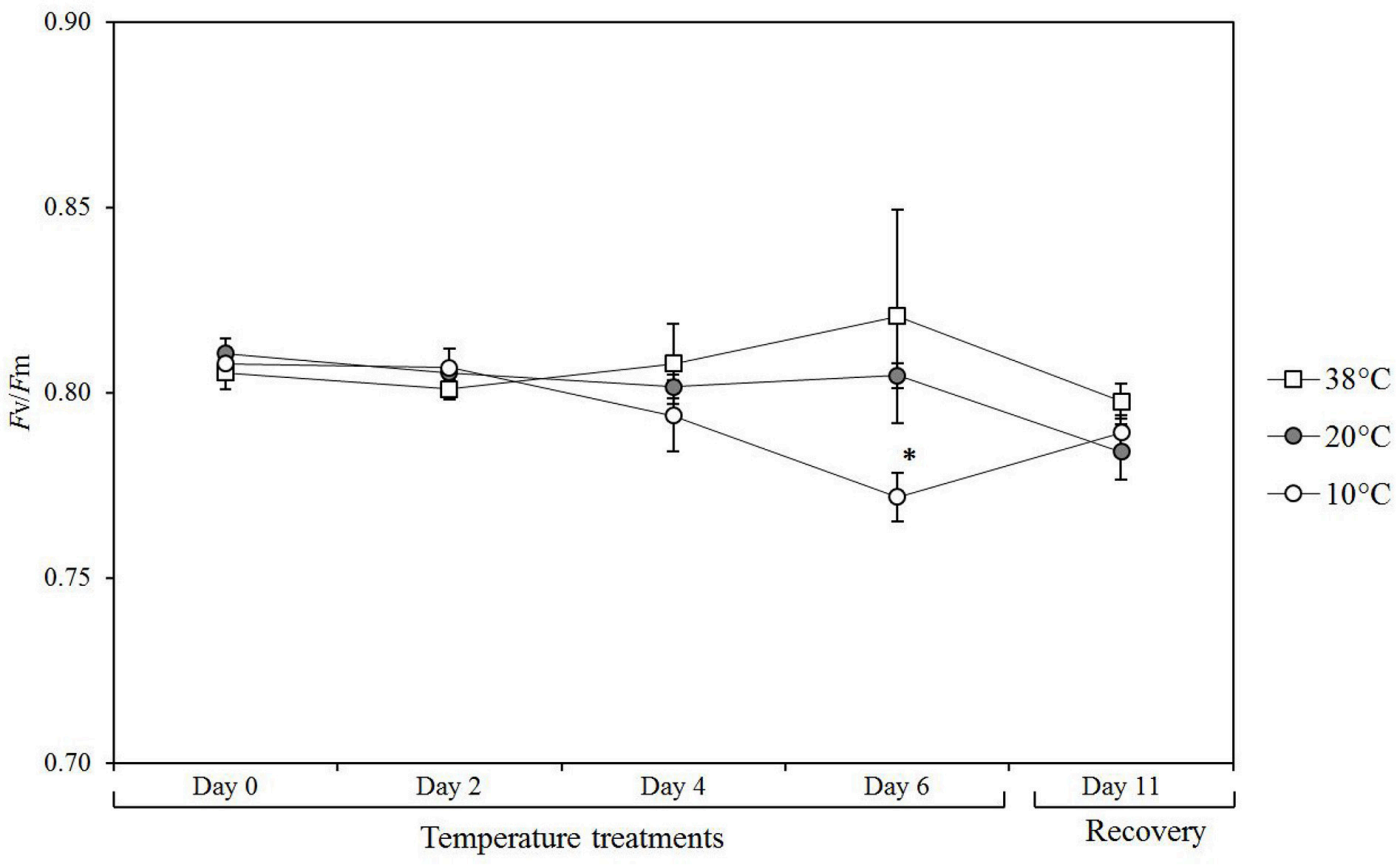
**Photosynthetic efficiency in tomato leaves over a time course of exposure to different temperatures**. Values are the mean of 5 biological replicates (*n* = 5) from plants exposed to 10, 38, and 20°C (control conditions) over 6 days followed by 5 days of recovery at control temperature up to day 11. Means ± SE. Significant differences in data between temperature treatments are indicated: ^*^P < 0.05, by One-Way ANOVA.

Chlorophyll fluorescence was measured to determine if prolonged exposure to varying temperatures (10°C moderate cold stress, 38°C heat stress, 20°C control condition) had an impact on the photosynthetic efficiency (Figure 1). Measurements of photochemical efficiency or quantum yield of photosystem II (*F*v/*F*m) values were carried at day 0 at 20°C and subsequently measured after 2, 4, 6 days, as well as after 5 days of recovery period at 20°C. 38°C did not have a significant effect over the period of treatment. The plants exposed to 10°C showed a slight but significant reduction in *F*v/*F*m (*p* = 0.002) after 6 days and recovered after 5 days at control conditions (20°C).

### Heat stress reduces chlorophyll content in tomato leaves

**Figure 2:**
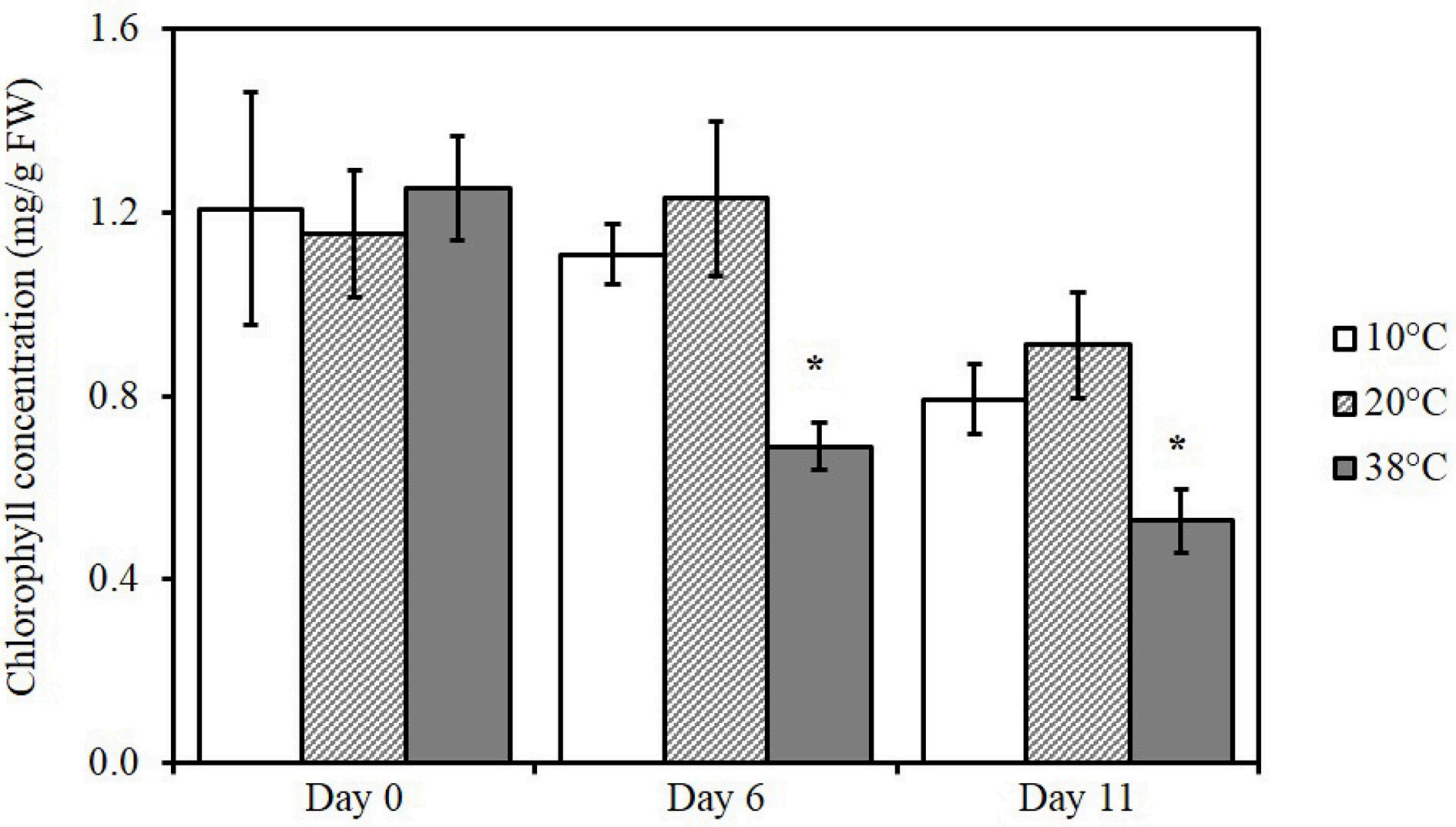
**Changes in chlorophyll content in tomato leaves exposed to different temperatures**. Means (*n* = 5) ± SE. Significant differences in data between temperatures are indicated: ^*^p < 0.05, by One-Way ANOVA, followed by Bonferroni–Holm, *post-hoc* test.

Total chlorophyll from tomato leaves was extracted and measured spectrophotometrically to determine differences in chlorophyll content after exposure to the three different temperatures (Figure 2). A significant reduction in total chlorophyll content was detected for tomato plants subjected to 38°C (*p* = 0.0107) for 6 days and after five additional days at 20°C for recovery (*p* = 0.0289; 11 days). Exposure to 10°C had no effect on chlorophyll levels.

### Untargeted lipidomics identify changes in lipid composition in tomato leaves after high temperature treatment

**Figure 3:**
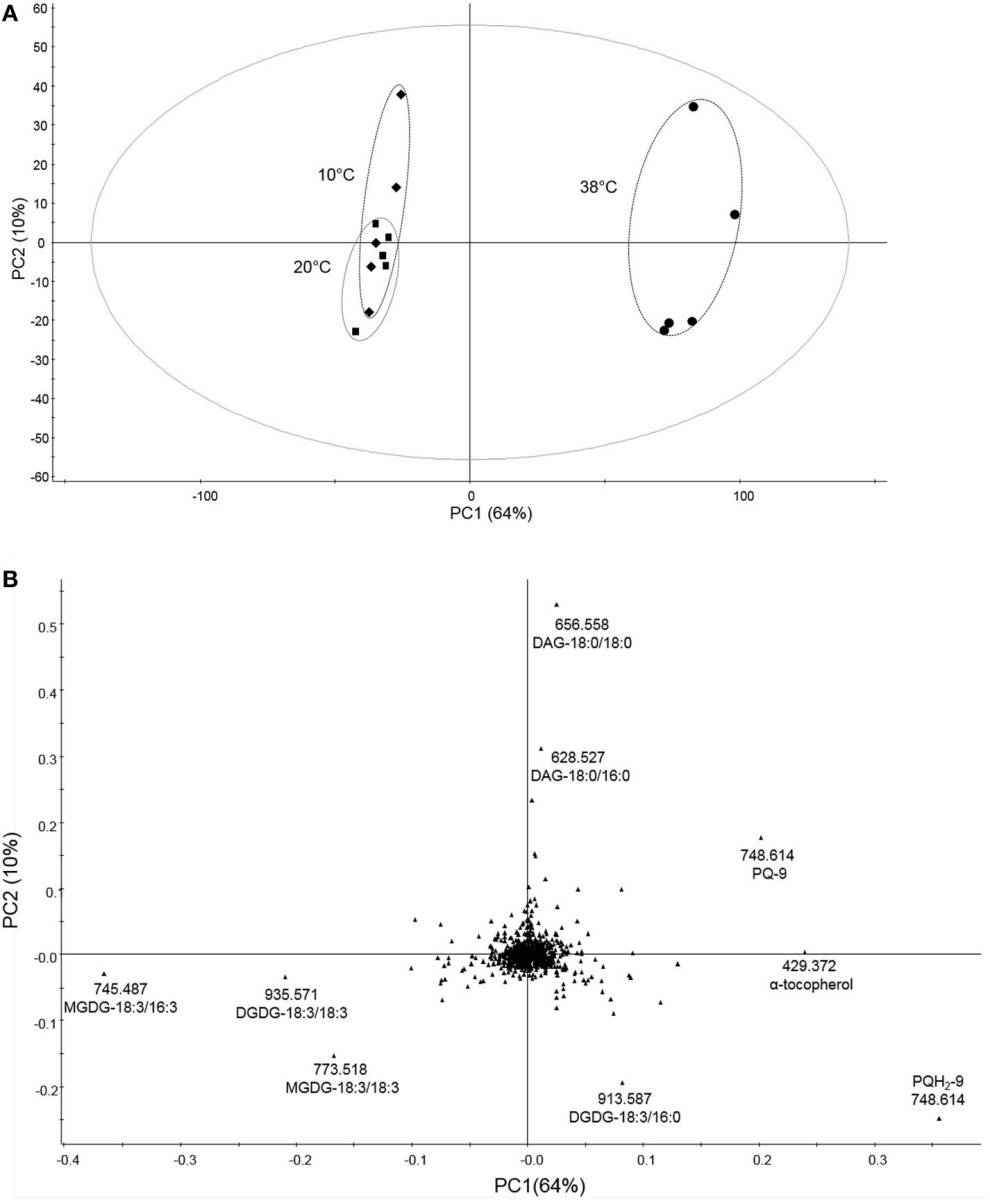
**Untargeted lipidomic profile of tomato leaves subjected to 6 days of low and high temperature treatments. (A)** Principal component analysis (PCA) of the lipid composition of leaf samples exposed to 10, 38, and 20°C (control conditions), over 6 days of temperature treatments. **(B)** Corresponding loading plots. Data were Pareto scaled prior to principal component analysis. PC1 and PC2 are first and second principal components, respectively, with their percentage of explained variance. Refer to Supplemental Table 1 for PCA loadings sorted according to the PC1.

To determine the differences in lipid composition after exposure to the three different temperatures, we carried out untargeted lipidomics analysis (Figure 3). The data obtained from total lipid extracts of leaves from each treatment were subjected to multivariate analysis to determine differences in lipid content between different temperatures. By this method, 791 markers were detected, most of which were not identified (Supplemental Table 1). A PCA model was established to reduce data complexity according to variation in lipid content found after temperature treatments. PCA identifies and ranks major sources of variance, which allows to cluster samples based on similarities and differences, in this case, in measured lipid profiles. PCA displayed two distinct clusters when comparing the variability of lipids followed by temperature treatment tested in five biological replicates each (Figure 3A). In the loadings plot (Figure 3B), the most contributive features of the first principal component (PC1) were selected and characterized by a combination of tandem mass spectrometry data and consultation of databases such as LIPID MAPS (Supplemental Table 1). This revealed that prenylquinones, namely plastoquinol (PQH_2_-9), plastoquinone (PQ-9), and α-tocopherol mostly contributed to the separation of the high temperature cluster, together with the galactolipid DGDG-18:3/16:0. Lower temperature treatment clusters were characterized by the presence of more unsaturated galactolipids such as MGDG-18:3/16:3 (Supplemental Figure 1), -18:3/18:3, and DGDG-18:3/18:3 (Figure 3B). Loadings located near the center of the plot, (i.e., the vast majority) make a negligible contribution to metabolic variation.

**Figure 4:**
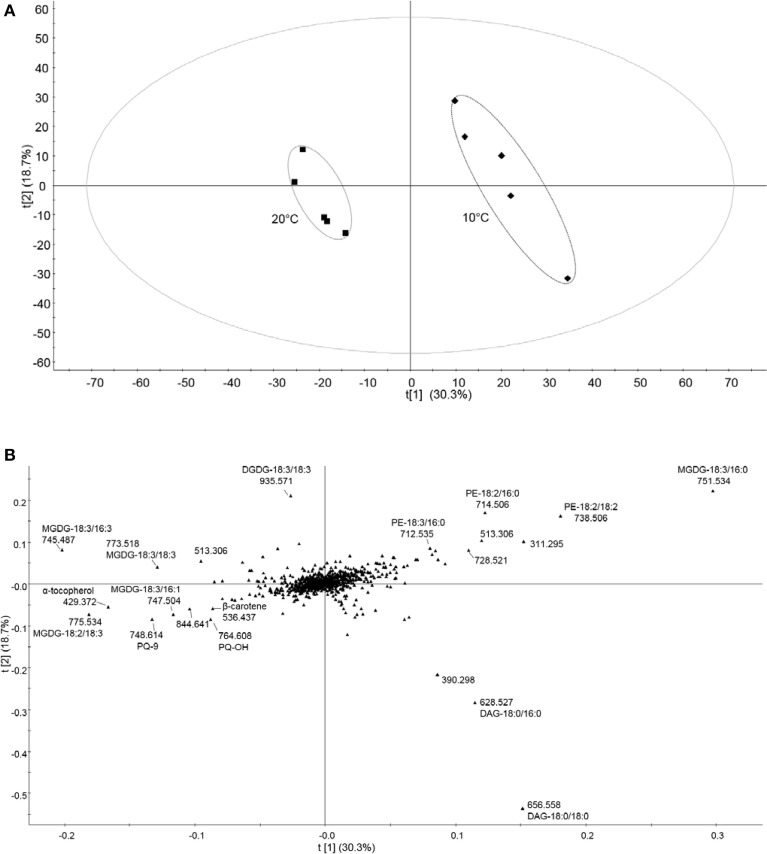
**Untargeted lipidomic profile of tomato leaves subjected to 6 days of low temperature treatment. (A)** Partial least squares discriminant analysis (PLS-DA) of the lipid composition of leaf samples exposed to 10 and 20°C (control conditions), over 6 days of temperature treatments. t[1] and t[2] are first and second latent variables, respectively, with their percentage of explained variance. Applying cross-validation on the data yielded high R2Y and Q2 coefficient values, which demonstrates the validity of the model (R2Y = 0.988, Q2 = 0.899). **(B)** Corresponding loading plots. Data were Pareto scaled prior to PLS-DA. Refer to Supplemental Table 1 for PLS-DA loadings sorted according to the PC1.

To evaluate variations between cold treatment and control conditions data were submitted to supervised PLS-DA. In contrast to PCA, which failed to separate the two treatments, PLS-DA revealed two distinct clusters (Figure 4A). Loadings from PLS-DA revealed that saturated diacylglycerols (DAG), such as DAG-18:0/18:0 and -18:0/16:0, phosphotidylethanolamines (PE), such as PE-18:2/18:2, -18:2/16:0, and saturated MGDG-18:3/16:0 were enhanced in the low-temperature samples (Figure 4B). Moreover, unsaturated galactolipids, such as MGDG-18:2/18:3, -18:3/16:3, -18:3/16:1 and DGDG-18:3/18:3, as well as prenylquinones such as α-tocopherol, PQH_2_-9, PQ-9, PQ-OH, and β-carotene contributed to the control treatment cluster (Figures 4A,B).

### High temperature increases prenylquinones in tomato leaves

**Figure 5:**
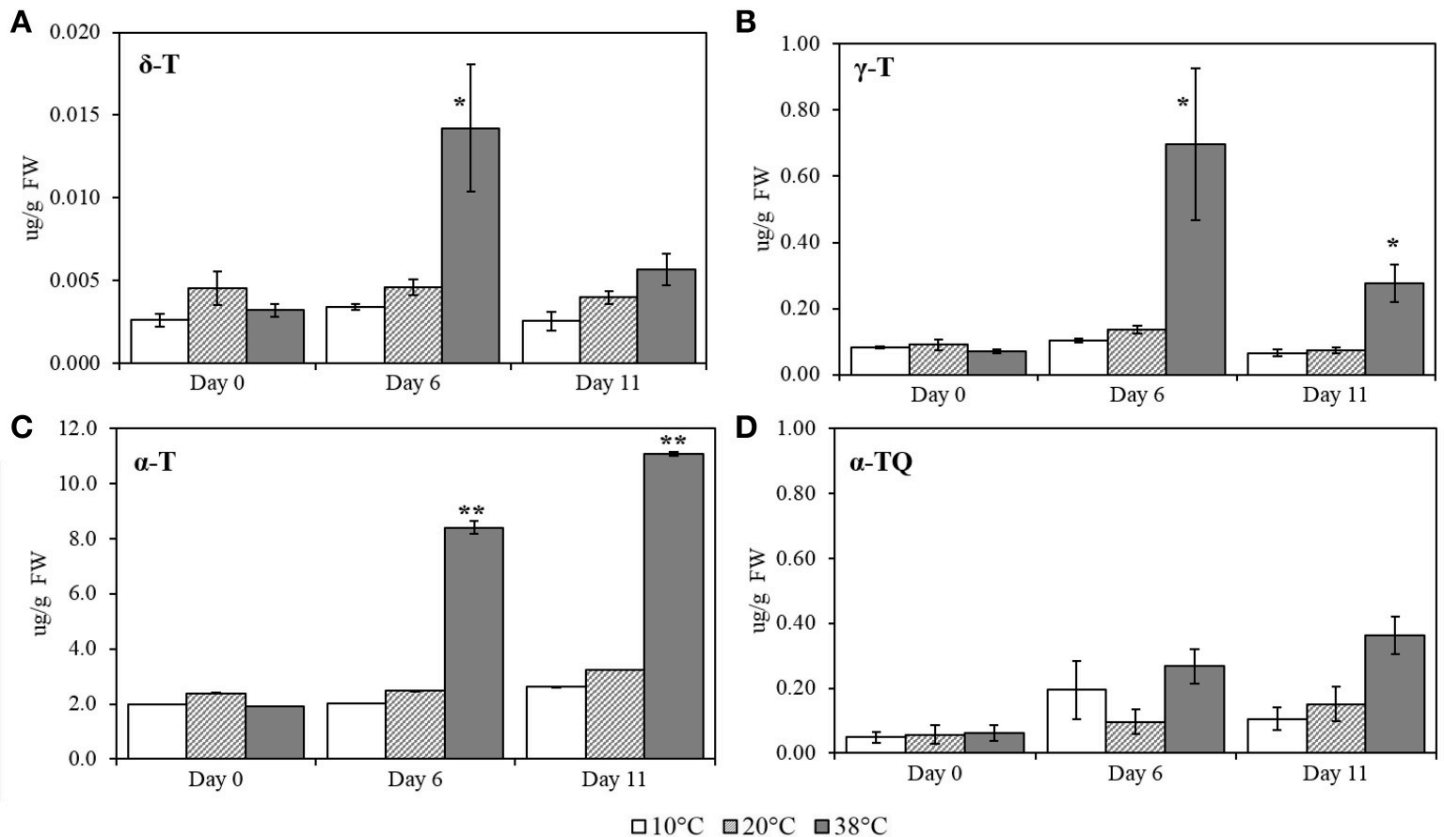
**δ-T (δ-tocopherol), γ- T (γ-tocopherol), α-T (α-tocopherol) and α-TQ (α-tocopherol quinone) quantification in tomato leaves after exposure to three different temperatures**. **(A)** δ-T; **(B)** γ-T; **(C)** α-T; **(D)** α-TQ. Lipids were extracted from plants submitted to 10, 38, and 20°C for 6 days and then allowed to recover at 20°C control temperatures (Day 11); values are means ± SE, (*n* = 5). Significant differences in data between temperatures are indicated: ^*^, *p* < 0.05 and ^**^, *p* < 0.001; by One-Way ANOVA, followed by Bonferroni-Holm, *post-hoc* test.

To quantify the effects of different temperatures on tocopherols, δ-T (δ-tocopherol), γ-T (γ-tocopherol), α-T (α-tocopherol), and α-TQ (α-tocopherol quinone) were measured, using pure standards (Figure 5). Levels of δ-T, γ-T (*p* = 0.013), and α-T (*p* < 0.001) increased under 38°C for Day 6. Levels of α-TQ, the oxidation product of α-T, appeared to increase at both 10 and 38°C, at Day 6, albeit not significantly when compared to control conditions. At Day 11, α-T (*p* < 0.001) and γ-T (*p* = 0.001) concentrations still remained significantly increased.

**Figure 6:**
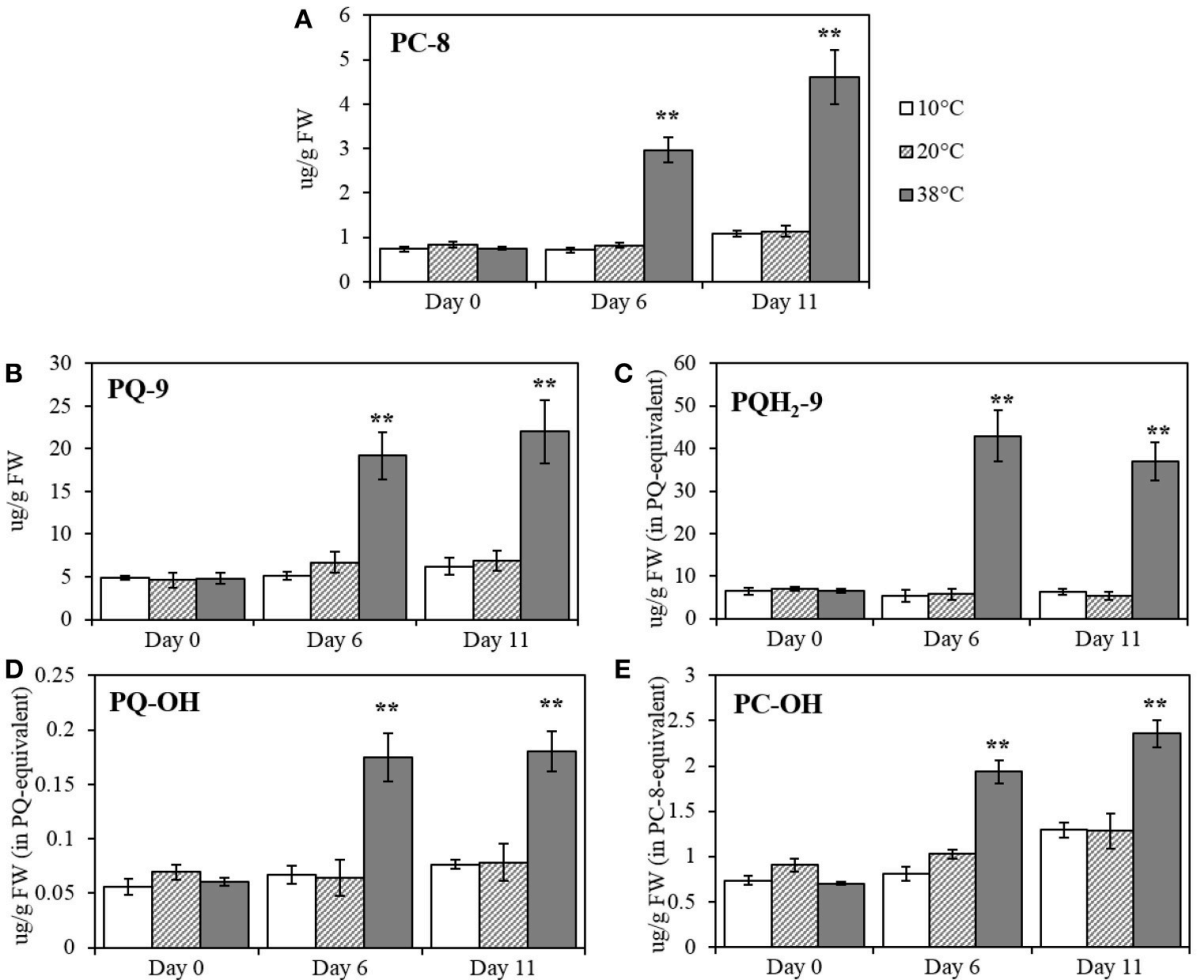
**PC-8 (plastochromanol), PQ-9 (plastoquinone), PQH_**2**_-9 (plastoquinol), PQ-OH (hydroxy-plastoquinone), and PC-OH (hydroxy-plastochromanol) quantification in tomato leaves after temperature treatment and recovery period**. **(A)** PC-8; **(B)** PQ-9; **(C)** PQH_2_-9; **(D)** PQ-OH; **(E)** PC-OH. Lipids were extracted from plants exposed to 10, 38, and 20°C for 6 days and then allowed to recover under 20°C control conditions for five additional days (Day 11); values are means ± SE, (*n* = 5). Significant differences in data between temperatures are indicated: ^*^, p < 0.05 and ^**^, p < 0.001; by One-Way ANOVA, followed by Bonferroni-Holm, *post-hoc* test.

**Figure 7:**
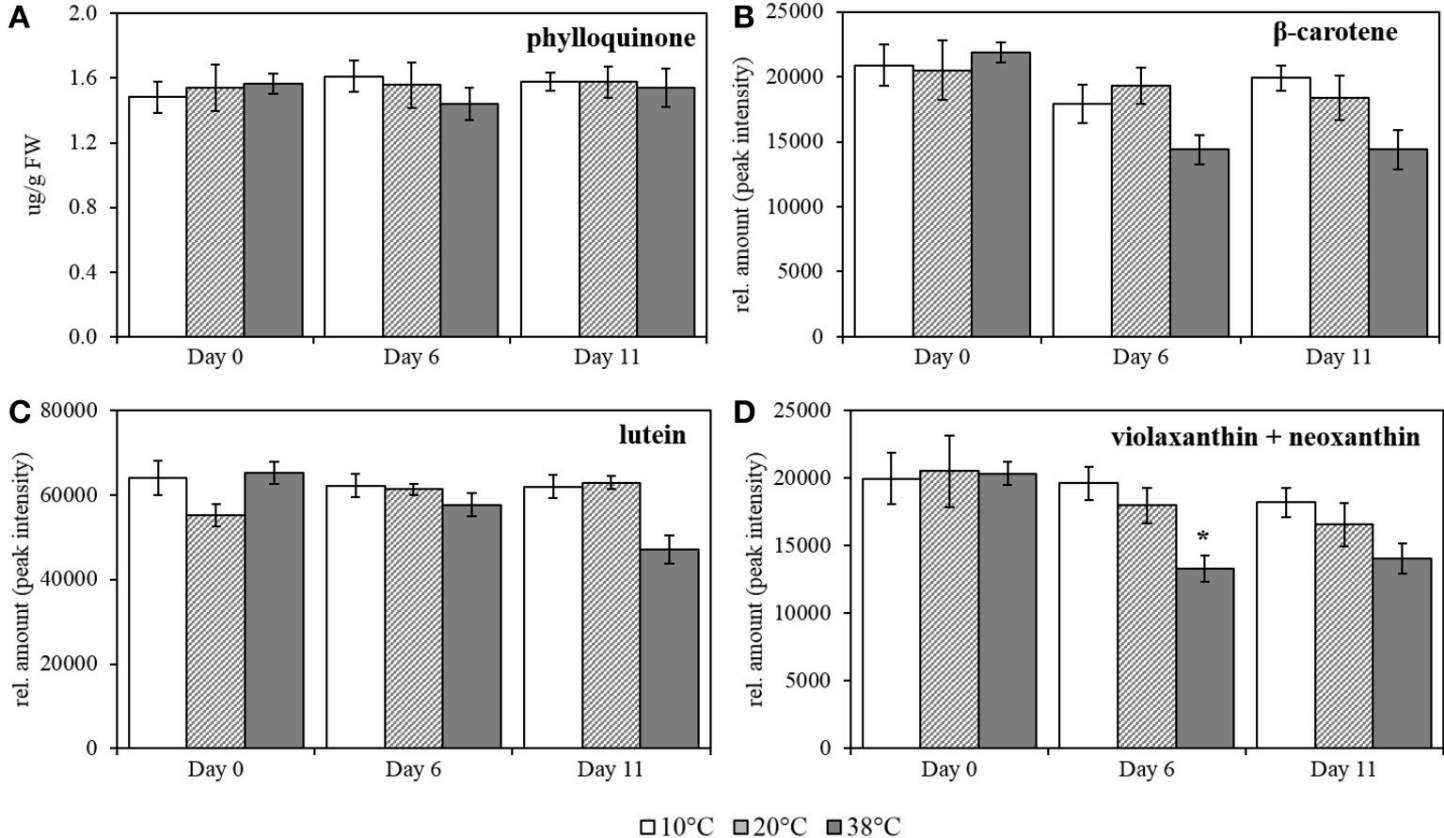
**Phylloquinone, β-carotene, lutein and combined violaxanthin+neoxanthin quantification in tomato leaves after temperature treatment and recovery period. (A)** phylloquinone; **(B)** β-carotene; **(C)** lutein, and **(D)** violaxanthin + neoxanthin. Lipids were extracted from plants exposed to 10, 38, and 20°C for 6 days and then allowed to recover under 20°C control conditions for five additional days, Day 11; values are means ± SE, (*n* = 5). Relative amount was calculated by peak intensity measurement in the chromatograms. Significant differences in data between temperatures are indicated: ^*^p < 0.05 and ^**^p < 0.001; by One-Way ANOVA, followed by Bonferroni-Holm, *post-hoc* test.

The effect of different temperatures on plastoquinol, plastochromanol, and their relatives, PQ-9 (plastoquinone), PQH_2_-9 (plastoquinol), PQ-OH (hydroxy-plastoquinone), PC-8 and PC-OH (hydroxyplastochromanol) were measured and quantified, using pure standards (Figures 6A–E). All of the compounds showed a significant increase at Day 6 at 38°C (*p* < 0.001; Figure 5) and remained significantly (p < 0.001) higher after five additional days of recovery under 20°C control conditions (Day 11). Phylloquinone content was quantified using a pure standard (Figure 7A) but no changes was detected under any of the temperatures tested.

High temperature provokes a slight decrease in content of carotenoids in tomato leaves. Carotenoid contents were quantified after exposure to the same temperature regime as for the prenylquinones. For β-carotene, lutein, violaxanthin, and neoxanthin together relative quantification was carried out by measuring the peak intensity in the chromatograms (Figures 7B–D; respectively). In contrast to the prenylquinones no significant changes in relative abundance of any of the compounds was observed under any of the three conditions tested except for combined violaxanthin and neoxanthin, which showed a significant decrease at Day 6 at 38°C (*p* = 0.007; Figure 7D).

### Temperature modulates saturation of galactolipids mostly present in thylakoid membranes

**Figure 8:**
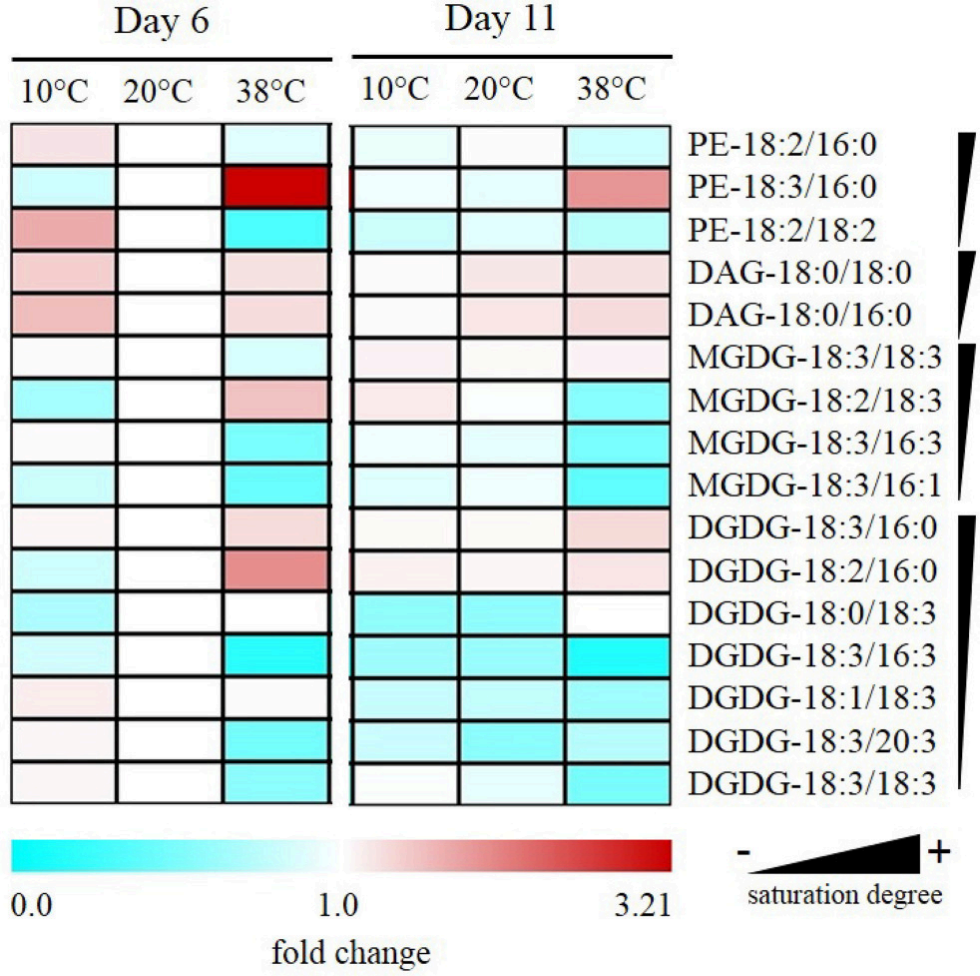
**Alteration of saturation levels of fatty acid-containing membrane lipids in tomato leaves under different temperatures**. Data are means (*n* = 5) and are expressed by fold change of fatty acid-derived lipids detected relative to control (20°C) treatment at Day 6. Phosphotidylethanolamine (PE); diacylglycerol (DAG); monogalactosyldiacylglycerol (MGDG); digalactosyldiacylglycerol (DGDG) are organized according to their saturation degree.

Fatty acid-derived lipids were profiled in tomato leaves exposed to the same temperature regime as for prenylquinones and carotenoids. Phosphatidylethanolamine (PE), diacylglycerol (DAG), MGDG, DGDG were organized according to their saturation degree (Figure 8) and identified following the *sn*-2 position of the glycerol backbone structure (prokaryotic referring to 16C fatty acid chains of plastid origin and eukaryotic to 18 C fatty acid chains of endoplasmic reticulum origin; Roughan and Slack, 1982). The DAG feeding chloroplast lipid synthesis originates from two separate pathways: the endoplasmic reticulum-localized eukaryotic pathway and the prokaryotic pathway that is located at the plastid inner envelope (Ohlrogge and Browse, 1995). Data are means of relative fold change of fatty acid-derived lipids detected relative to control (20°C) treatment at Day 6.

After 6 days at 38°C, there was a decrease in levels of unsaturated PE-18:2/18:2 accompanied by an increase in PE-18:3/16:0. Also, there was a reduction of unsaturated MGDGs of prokaryotic origin such as MGDG-18:3/16:3 and MGDG-18:3/16:1. Moreover, there was also a significant reduction in unsaturated DGDG-18:3/16:3 together with an increase in saturated DGDG-18:3/16:0 and -18:2/16:0. In fatty acid-derived lipids of eukaryotic origin, alterations of abundance were less remarkable but still, there was a significant reduction in the abundance of highly unsaturated DGDG-18:3/18:3 and -18:3/20:3 together with an increase of MGDG-18:2/18:3 under high temperature. In contrast to high temperature exposure, MGDG-18:2/18:3 significantly decreased after low-temperature exposure, and PE-18:2/18:2 increased significantly contrasting with its decrease at high temperature.

No significant changes were observed at the different temperatures for PE-18:2/16:0; DAG-18:0/18:0 and -18:0/16:0, nor for the eukaryotic MGDG-18:3/18:3, -18:2/18:3, and DGDG-18:1/18:3 and -18:0/18:3.

## Discussion

Climate change exposes plants to increasing temperatures. Changes in temperature impact plant physiology with potentially deleterious effects. Inhibition of photosynthesis at the level of Photosystem II has been reported to occur after short exposure to high temperature (35–40°C), in a variety of species (Havaux, 1993; Law and Crafts-Brandner, 1999; Crafts-Brandner and Law, 2000; Crafts-Brandner and Salvucci, 2002). Changes in temperature also have a direct influence on the physical properties of thylakoid membranes and affect electron transport dynamics (Williams, 1998). In this study, we aimed to determine the dynamics of lipid metabolism in response to high temperature (38°C) treatment and its impact on the photosynthetic efficiency of tomato plants using moderately low temperature (10°C) and 20°C as a reference. In contrast to other studies, we did so at a lipidome-wide level and used statistical analysis to determine the most important changes. This approach allows to put observed changes in a wider context and gain insight on the relative importance of the observed effects. The ability of the plants to acclimate to the temperature regimes was probed by chlorophyll fluorescence measurements that give a measure of photosynthetic efficiency (Sinsawat et al., 2004) as well as photoinhibition at the level of Photosystem II that is the most thermosensitive component of the photosynthetic membrane (Berry and Björkman, 1980; Srivastava et al., 1997; Mathur et al., 2014). Chlorophyll contents was also measured as it is known to be reduced by high temperature stress (Kumar Tewari and Tripathy, 1998; Dutta et al., 2009; Efeoglu and Terzioglu, 2009). Indeed, high temperature (38°C) treatment also reduced the chlorophyll contents in tomato (Figure 2). However, photosynthetic efficiency *F*v/*F*m was not reduced over the duration of the experiment at 38°C suggesting that the plants acclimated well. In contrast, 10°C treatment led to a reduction of *F*v/*F*m by around 4%. This mild photoinhibition suggested that tomato plants do not acclimate as well to low temperatures as to high temperatures.

Using non-targeted lipidomics, we detected 791 molecular species in the mass spectra of total tomato leaf extracts. To determine the most important differences between the three conditions we used principle component analysis followed by the identification of compounds combining mass spectrometric information with searches in the online databases. The first principal component was dominated by the presence of prenylquinones and membrane lipids. These results indicate, that the most important changes at the lipidome-wide level occur in tocopherols, plastoquinone/plastoquinone as well as their metabolites (Figure 4) and in the degree of fatty acid saturation of galactolipids.

Several studies have already demonstrated that tocopherol production correlates with oxidative stress (for instance under high-light conditions), and suggested that this may reflect the ability of tocopherols to quench ROS and protect Photosystem II (Havaux et al., 2005; DellaPenna and Pogson, 2006; Krieger-Liszkay and Trebst, 2006; Kobayashi and DellaPenna, 2008). Tocopherol biosynthesis is dependent on the availability of phytol. Recently, it has been demonstrate that phytyl diphosphate originating from chlorophyll degradation contributes significantly to tocopherol biosynthesis (Almeida et al., 2015; vom Dorp et al., 2015). It is therefore possible that the chlorophyll degradation observed at 38°C contributes directly to the increased biosynthesis of tocopherols under this condition. A temperature-dependent increase on the kinetics of prenylquinone biosynthesis at 38°C cannot be excluded. However, the biosynthesis of phylloquinone as well as that of vast majority of other compounds in the samples were not affected. This suggests, that there is no sweeping Arrhenius effect that would widely upregulate the synthesis of many lipid compounds (Kaplan et al., 2004; Guy et al., 2007; Ruelland and Zachowski, 2010). The high concentrations of tocopherols produced under high temperature, much like high-light, can probably be rationalized by an increased requirement for lipid antioxidants in reponse to this stress. A recent study reported a seven-fold increase in α-tocopherol in soybean that was grown at moderately high temperatures (33/25°C; Chennupati et al., 2011). Tocopherol levels in leaves subjected to 10°C treatment (in contrast to high temperature) were not significantly different from tocopherol levels detected under control temperature.

The decline in *F*v/*F*m in plants subjected to 10°C treatment suggests that the constant concentrations of the major prenylquinones at this temperature may be insufficient for plants to cope with this condition and, therefore, lead to photoinhibition. However, the decline in *F*v/*F*m was reversible indicating that no permanent damage had been inflicted (Haldimann et al., 1996). Low temperatures slow down enzymatic reactions and interfere with both antioxidant biosynthesis and regeneration leading to inhibition of photosynthesis (Jahnke et al., 1991; Allen and Ort, 2001). This may also pertain to tocopherol and explain why photoinhibition is inflicted. The unchanged concentrations of tocopherols under low-temperature treatment may also contribute to the destabilization of biophysical properties, such as the fluidity of the thylakoid membrane (Hincha, 2008).

Plastoquinone and plastoquinol were the most increased molecular species under high temperature. Plastoquinone is best known as an electron carrier in the photosynthetic electron transport chain (Amesz, 1973). However, recent reports identify plastoquinone as a lipid antioxidant functioning as a scavenger of singlet oxygen species and inhibitor of lipid peroxidation (Olejnik et al., 1997; Gruszka et al., 2008; Kruk and Trebst, 2008; Mène-Saffrané et al., 2010; Szymañska and Kruk, 2010; Nowicka and Kruk, 2012; Nowicka et al., 2013; Kruk et al., 2014). A recent report showed that an Arabidopsis line overexpressing a gene in the biosynthetic pathway of PQ-9 SPS1 (SOLANESYL DIPHOSPHATE SYNTHASE 1) had two- to three-fold higher levels of PQ-9 and was more resistant to photooxidative stress under excess light when compared to the wild type, showing a decrease of bleaching, lipid peroxidation, and PSII photoinhibition (Ksas et al., 2015). Barley seedlings exposed to 3 h of high temperature treatment showed a reduction in the size of the photoactive plastoquinone-pool present in thylakoids, suggesting a transfer to the non-photoactive pool under temperature stress conditions (Pshybytko et al., 2008). The non-photoactive plastoquinone pool is not active in the photosynthetic electron transport chain but participate in anti-oxidant reactions. The non-photoactive pool is contained in the plastoglobules (Szymañska and Kruk, 2010; Ksas et al., 2015). Our results showed a massive accumulation of PC-8, PQH_2_-9, and PQ-9 as well as its oxidized metabolites PC-OH and PQ-OH (Figure 5) under high temperature. It appears likely that the increase of PQ and metabolites under high temperature reflect the role of PQH_2_-9 as a powerful membrane antioxidant rather than that of an electron carrier. Therefore, the increase of PQH_2_-9 and PQ-9 may contribute mostly to the non-photoactive pool that is located in thylakoid-bound plastoglobules lipid droplets (Kruk and Trebst, 2008; Ksas et al., 2015). Carotenoids are important components in the light harvesting complexes, but have other functions, including the xanthophyll cycle in NPQ to dissipate excess excitation energy (DellaPenna, 1999; Müller et al., 2001). The interaction of carotenoids with membrane lipids may also directly influence membrane physical properties (Havaux, 1998). When carrying out targeted analysis of carotenoids, the only significant change we observed was a reduction of the sum of violaxanthin and neoxanthin after 6 days exposure to 38°C. It thus appears, that resistance to high temperatures does not require increased levels of carotenoids in general and that the decrease observed for xanthophylls does not have deleterious effects on NPQ and the ability to dissipate excess excitation energy.

Membrane fluidity is a function of the degree of saturation of membrane fatty acid lipids and temperature (Zheng et al., 2011). Our results confirm in tomato leaves that fatty acid saturation decreases at high temperatures and increases in the cold and show that these are amongst the most important lipidome-wide changes occurring. The most significant differences concern phosphatidylethanolamine (PE), an extraplastidic phospholipid, and mono- as well as digalactosyldiacylglyerol. Unsaturated PE-18:2/18:2 increased significantly at low-temperature contrasting with its decrease at high temperature. The prokaryotic origin galactolipids rich in unsaturated fatty acids, MGDG-18:3/16:3, -18:3/16:1 as well as DGDG-18:3/16:3, exhibited a striking drop at high temperature when compared to control and low temperature. In contrast, levels of more saturated DGDG-18:3/16:0 and -18:2/16:0 increased under high temperature treatment. These results are in agreement with reports that have shown an increase of the degree of saturation of fatty acids in Arabidopsis exposed to 36°C when compared to plants grown at 17°C (Falcone et al., 2004). The increase of saturation in membrane lipids has been shown to confer thermotolerance to Arabidopsis and tobacco plants (Kunst et al., 1989; Murakami et al., 2000; Routaboul et al., 2012).

Several unsaturated membrane lipids of eukaryotic origin decreased significantly under low temperatures, possibly because they are substrates of eukaryotic DGDG formation. The increase of unsaturated MGDG-18:2/18:3 at high temperature could be explained by a possible high temperature-induced perturbation affecting its recycling into DGDG. It has been reported, that lipid saturation levels increase over time under heat stress, implying that there is a relation between the degree of saturation of leaf membrane prior to heat stress and the ability of that plant to limit heat-induced damages during the stress period (Larkindale and Huang, 2004).

## Author contributions

FK and LS designed the research. LS carried out the experimental work and statistical analysis. GG developed the UHPLC-APCI-QTOFMS method, conducted measurements, identified compounds and critically read the manuscript. LS and FK wrote the manuscript.

### Conflict of interest statement

The authors declare that the research was conducted in the absence of any commercial or financial relationships that could be construed as a potential conflict of interest.

## Supplementary material

The raw data used in this paper is archived at the Plant and Microbial Metabolomics Resource (PRM; URL: “http://www.metnetdb.org/PMR/experiments/?expid=259”).
