# Supplementary material for: Lipid Antioxidant and Galactolipid Remodeling under Temperature Stress in Tomato Plants
Source: Front Plant Sci. 2016 Feb 17;7:167. doi: 10.3389/fpls.2016.00167 (PMC4756161; doi:10.3389/fpls.2016.00167)

## Slide 1
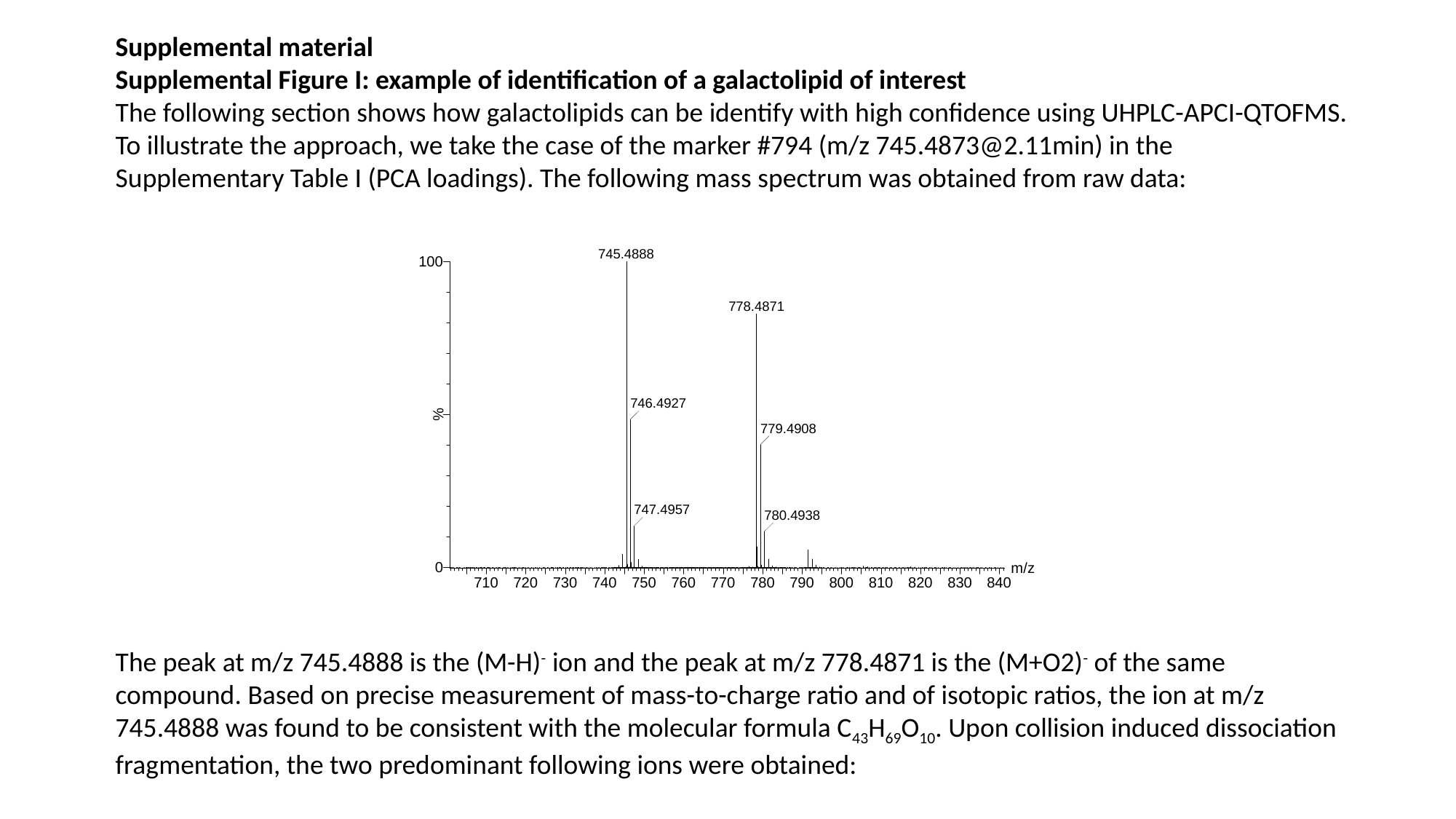

Supplemental material
Supplemental Figure I: example of identification of a galactolipid of interest
The following section shows how galactolipids can be identify with high confidence using UHPLC-APCI-QTOFMS. To illustrate the approach, we take the case of the marker #794 (m/z 745.4873@2.11min) in the Supplementary Table I (PCA loadings). The following mass spectrum was obtained from raw data:
The peak at m/z 745.4888 is the (M-H)- ion and the peak at m/z 778.4871 is the (M+O2)- of the same compound. Based on precise measurement of mass-to-charge ratio and of isotopic ratios, the ion at m/z 745.4888 was found to be consistent with the molecular formula C43H69O10. Upon collision induced dissociation fragmentation, the two predominant following ions were obtained:

## Slide 2
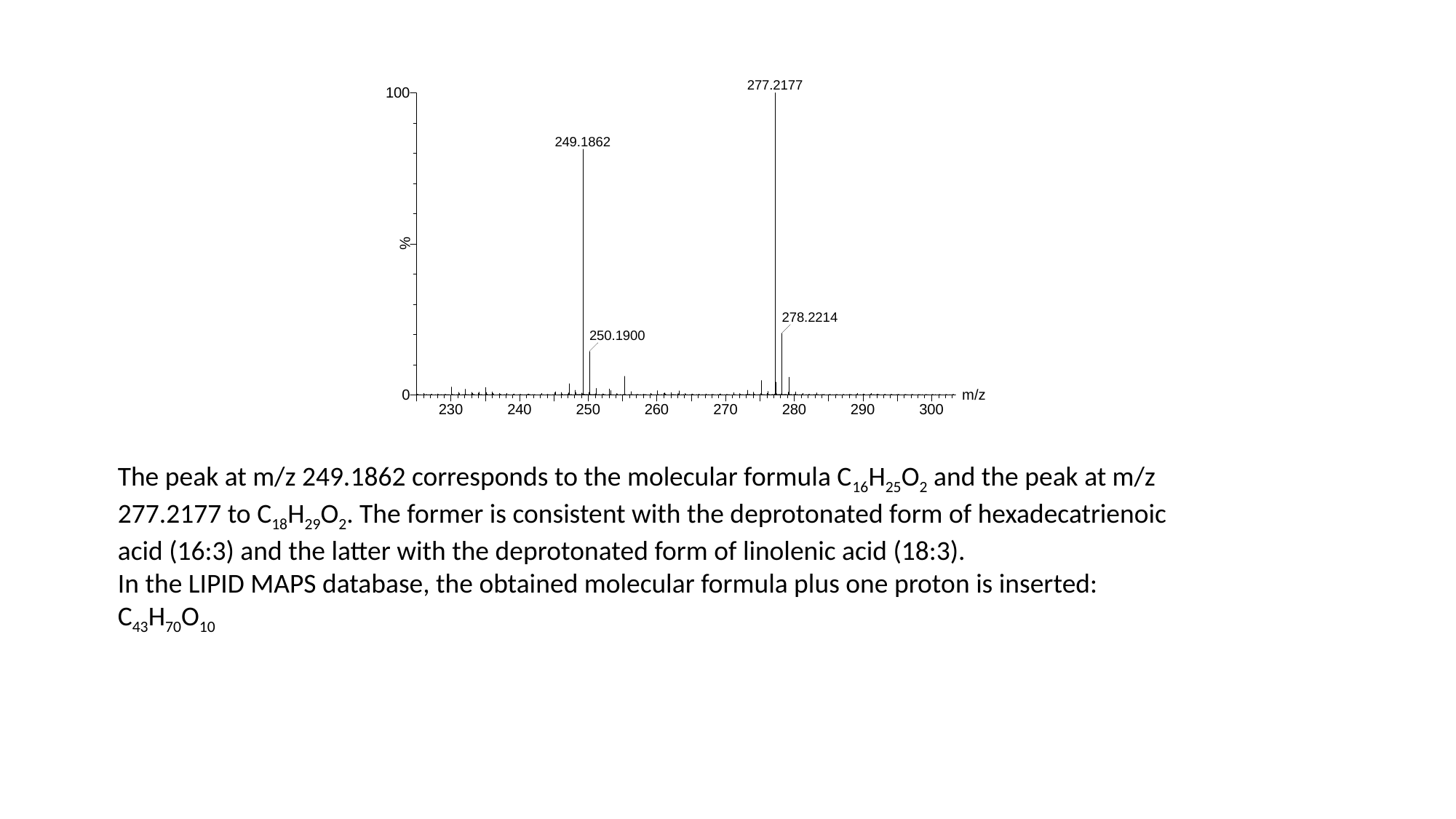

The peak at m/z 249.1862 corresponds to the molecular formula C16H25O2 and the peak at m/z 277.2177 to C18H29O2. The former is consistent with the deprotonated form of hexadecatrienoic acid (16:3) and the latter with the deprotonated form of linolenic acid (18:3).
In the LIPID MAPS database, the obtained molecular formula plus one proton is inserted: C43H70O10

## Slide 3
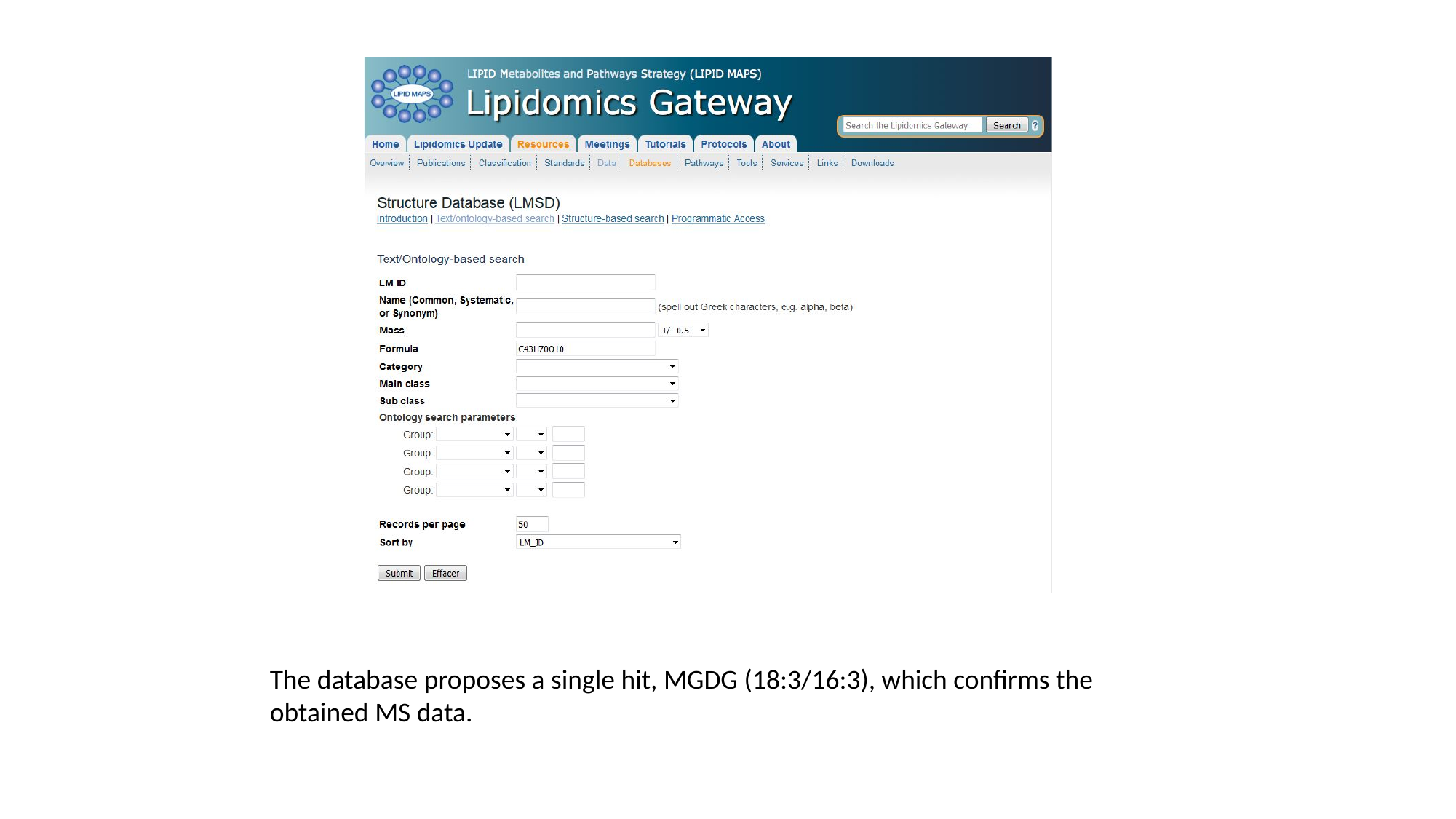

The database proposes a single hit, MGDG (18:3/16:3), which confirms the obtained MS data.

## Slide 4
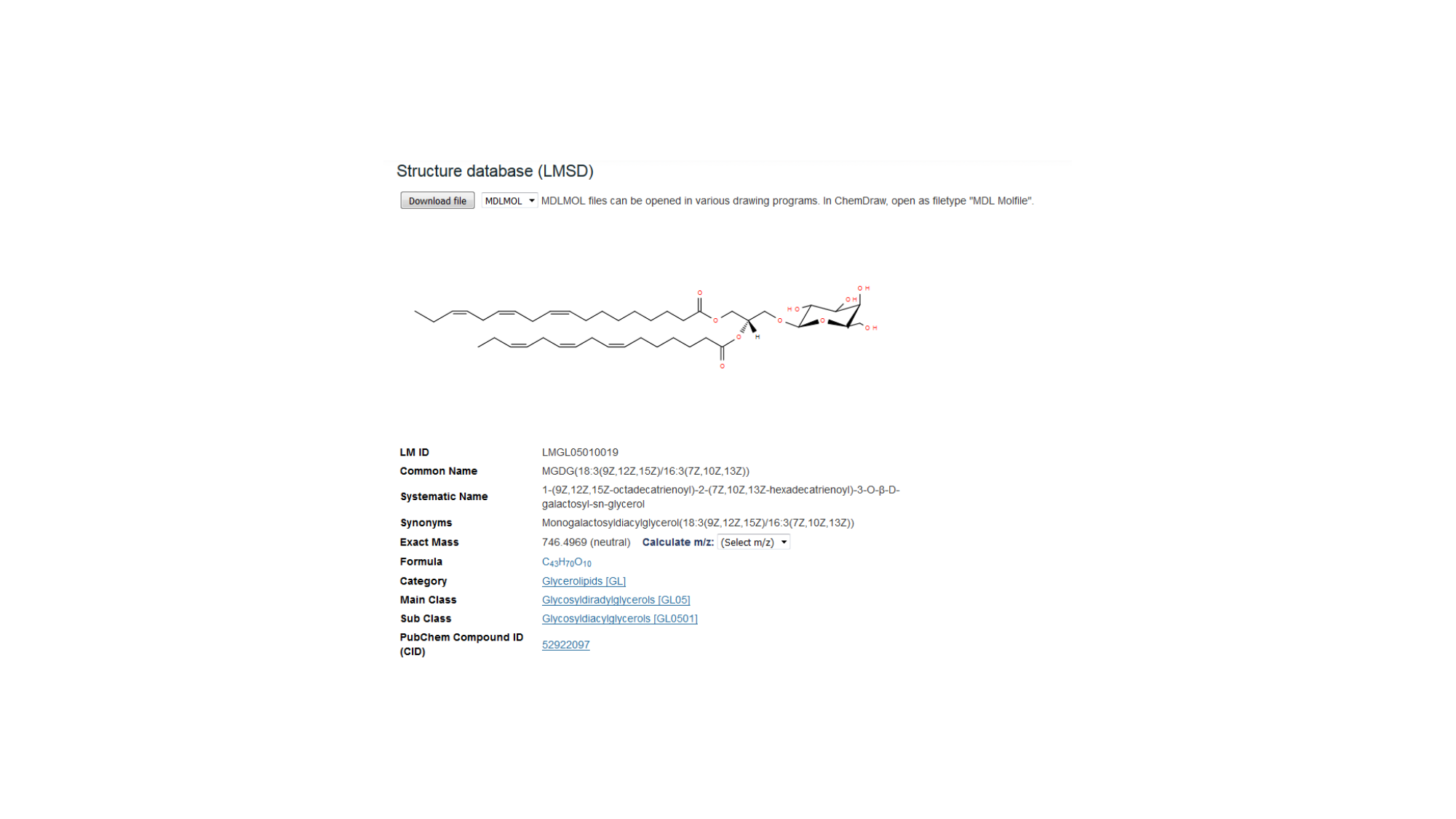

Supplement: Supplementary file 1 [file Presentation1.PPTX]
